# Community engagement and ethical global health research

**DOI:** 10.1080/11287462.2019.1703504

**Published:** 2019-12-20

**Authors:** Bipin Adhikari, Christopher Pell, Phaik Yeong Cheah

**Affiliations:** a Mahidol-Oxford Tropical Medicine Research Unit, Faculty of Tropical Medicine, Mahidol University, Bangkok, Thailand; b Centre for Tropical Medicine and Global Health, Nuffield Department of Medicine, Churchill Hospital, Oxford, UK; c Kellogg College, University of Oxford, Oxford, UK; d Centre for Social Science and Global Health, University of Amsterdam, Amsterdam, The Netherlands; e Amsterdam Institute for Global Health and Development, Amsterdam, The Netherlands; f The Ethox Centre, Nuffield Department of Population Health, University of Oxford, Oxford, UK

**Keywords:** Community engagement, ethics, research ethics, global health

## Abstract

Community engagement is increasingly recognized as a critical element of medical research, recommended by ethicists, required by research funders and advocated in ethics guidelines. The benefits of community engagement are often stressed in instrumental terms, particularly with regard to promoting recruitment and retention in studies. Less emphasis has been placed on the value of community engagement with regard to ethical good practice, with goals often implied rather than clearly articulated. This article outlines explicitly how community engagement can contribute to ethical global health research by complementing existing established requirements such as informed consent and independent ethics review. The overarching and interlinked areas are (1) respecting individuals, communities and stakeholders; (2) building trust and social relationships; (3) determining appropriate benefits; minimizing risks, burdens and exploitation; (4) supporting the consent process; (5) understanding vulnerabilities and researcher obligations; (6) gaining permissions, approvals and building legitimacy and (7) achieving recruitment and retention targets.

## Introduction

Community engagement is increasingly recognized as a critical element of health-related research, recommended by ethicists, required by funders and advocated in ethics guidelines, such as those issued in 2016 by the Council for International Organization of Medical Sciences (CIOMS) (CIOMS: International Ethical Guidelines for Health-Related Research Involving Humans, 2016; Dickert & Sugarman, 2005; King, Kolopack, Merritt, & Lavery, 2014). However, the term *community engagement* is interpreted in different ways across the domains of health promotion, health-related research and policy-making (Tindana et al., 2007). In part, this results from a varied understanding of the concepts “community” and “engagement” (Marsh, Kamuya, Parker, & Molyneux, 2011; Wilkinson, Parker, Martineau, & Leach, 2017).

Some definitions of *community* focus on shared locality, religion or race (Clinical and Translational Science Award [CTSA]), and others, particularly in health-related research, on common health problems or disease endemicity (Marsh et al., 2011). A broader definition, which is particularly relevant for research-related engagement, emphasizes how communities are collections of individuals with interests or a stake in the conduct and/or outcomes of a study and/or the interactions with the research team (King et al., 2014). Along these lines, in this article, community is considered to include the residents of settlements where health research is conducted, potential study participants, all other residents in the immediate locality and stakeholders from outside the area, including the ministries of health, public healthcare authorities, local research institutes and researchers.

Engagement occurs along a spectrum: from reaching out and informing, to consulting, involving, collaborating and sharing leadership (CTSA). Engagement therefore denotes a wide variety of activities, such as patient and public involvement events (e.g. science cafes events), consultation regarding study activities through community advisory boards (Cheah et al., 2010; Kamuya, Marsh, Kombe, Geissler, & Molyneux, 2013), participatory community drama (Lim, Peto, Tripura, & Cheah, 2016; Nguon et al., 2018) and participatory visual methods (Black, Davies, Iskander, & Chambers, 2018;; Marsh et al., 2011 O’Donovan et al., 2019). A specific engagement strategy – and associated activities – is contingent on the nature of the research it accompanies. For instance, engagement linked to a qualitative study examining HIV-related stigma would look quite different to the activities that accompany a large-scale randomized vaccine trial. Social and political dynamics also play a role in the nature of engagement (Adhikari et al., 2019): for example, power differences between the research team and the community (and within communities) may affect engagement processes and outcomes (Angwenyi et al., 2014).

Global health researchers often associate the aims of community engagement with achieving study goals, particularly in terms of promoting study recruitment and retention (Adhikari et al., 2016). Particularly in low-income contexts, economic, educational and power disparities between study staff and participants can contribute to suspicion in communities and to study refusals and withdrawls (Lavery, 2004; Morin et al., 2008; Newman et al., 2015). This can lead to premature study closure, and inadequate sample size which compromises researchers’ ability to generate high-quality evidence (Dickert & Sugarman, 2005; Mills et al., 2005; Wilkinson et al., 2017). Poverty, malnutrition, lack of health care infrastructure and high disease burden bring additional challenges (Lavery, 2004; MacQueen, Bhan, Frohlich, Holzer, & Sugarman, 2015). For instance, researchers might be obliged – prompted by demands from communities, or professional or personal responsibility – to address the burden of disease that is unrelated to the area of research, which potentially diverts resources away from achieving study aims.

Less emphasis has been placed on the value of community engagement with regard to ethical good practice, with goals often implied rather than clearly articulated. With a view to reaching engagement practitioners and researchers working in the field, this article outlines the potential contribution of community engagement to ethical global health research. To illustrate the various ways this can occur, we draw on a scoping literature review and our experience of conducting and evaluating a range of projects in the Greater Mekong Subregion. With community engagement programmes encompassing varying activities, we focus on what community engagement *can* achieve, rather than what it does achieve, and outline some of the barriers to achieving these goals.

## Goals of community engagement

### Respecting communities

Respecting communities entails acknowledging, informing, explaining to, consulting, listening to and/or collaborating with those who have a stake in the research or programme. At its most superficial, *acknowledging* the community can merely mean introducing the research team to community members (CTSA). *Informing* and *explaining* a study to communities requires providing information about the researcher, the research institutions, the study concept and rationale in appropriate formats (Adhikari et al., 2016). Appropriate formats can blend arts and education, using pictorial descriptions and videos (Adhikari et al., 2017; Lim et al., 2016; Nguon et al., 2018). *Consulting or listening to* community members entails researchers seeking their input, for example, through community advisory boards, about suitable ways to recruit study participants, to implement the study and to act appropriately as guests in the community (Cheah et al., 2010). Creative participatory methods, such as forum theatre and visual methods offer alternative ways to gain insights from communities compared to traditional methods, such as formal interviews or focus group discussions (Black et al., 2018; Boal & McBride, 1985 Wallerstein & Duran, 2010;). *Collaboration* between researchers and communities means that communities are involved as partners, co-develop the study protocol and share responsibilities in conducting research (Adhikari et al., 2017; Emanuel, Wendler, Killen, & Grady, 2004; National Institute for Health Research, 2012).

In practice, researchers are often constrained by funders’ priorities and a pre-determined set of study objectives, which can limit collaboration with communities (Lavery, 2018). In addition, budgets and protocols are often finalized with little room for reallocation of funds or additional activities. Collaboration may also be hampered if communities cannot contribute fully to decision-making, for example, if faced with an unfamiliar and extremely complex study rationale (Koen, Essack, Slack, Lindegger, & Newman, 2013). Because engagement often builds and relies on existing social hierarchies, power differentials may contribute to unintended consequences, such as subtle subservience and unquestioning acquiescence, sometimes, with authorities’ research agendas and even their personal interests (Angwenyi et al., 2014; Marsh et al., 2011). Close attention must therefore be paid to community power dynamics and in some instances may warrant limiting the involvement of authorities.

### Building trust and social relationships

Trust plays a critical role in the enrolment decisions of potential study participants. When faced with challenging scientific concepts, a complex study rationale and uncertain benefits, potential participants often rely on the extent to which they trust study staff or the research institution(s) involved (Geissler, Kelly, Imoukhuede, & Pool, 2008). In low-income settings, there are often significant disparities between researchers and participants in terms of education, income and familiarity with the scientific concepts that underpin a study design (Kajeechiwa et al., 2016; Lavery, 2004; Newman et al., 2015). These differences can prompt suspicions about researchers’ motives and who benefit most from the research, which can foster resentment and opposition (Lavery, 2004). Such responses are also influenced by local idioms, including those that describe the “stealing” of blood. Such “rumours”, often entwined with experiences of colonialism, neo-colonialism and exploitation, are sometimes drawn on to articulate apprehension about study procedures (Geissler & Pool, 2006; White, 2000). Often the short and managed interactions with study staff cannot build the trust needed to overcome such concerns (Geissler et al., 2008; Newman et al., 2015). Trust stems from the relationship between two or more individuals and/or between an individual and an institution (Molyneux, Peshu, & Marsh, 2005).

In practice, in contexts where health systems are weak, providing (quality) health care can help to build institutional trust (Geissler et al., 2008; Molyneux et al., 2005; Pratt et al., 2013). This has been described in the cases of established research institutions, such as the Kenya Medical Research Institute (KEMRI) in Kenya, the Medical Research Council (MRC) in The Gambia and the Shoklo Medical Research Unit (SMRU) in Thai-Myanmar border region, which provide health care in addition to leading research (Geissler et al., 2008; Molyneux et al., 2005; Pratt et al., 2013). Past interactions with institutions (particularly the staff who represent them) and the health benefits they have received influence the development of institutional trust. In such contexts, challenges can arise if community members struggle to distinguish between health care and research leading to “therapeutic misconception” (Appelbaum, Anatchkova, Albert, Dunn, & Lidz, 2012). Scholars however argue that in communities with minimal health facilities, providing care in appreciation of or in exchange for participation in research is often practical and justifiable (Geissler et al., 2008).

Inter-personal trust can be fostered through building relationships between community members and research staff, which are built over time through day-to-day interactions, sharing of food and participating in local events (Adhikari et al., 2018; Adhikari et al., 2019; Geissler et al., 2008; Sahan et al., 2017). In contexts where reciprocity and conformity are social norms, these relationships can inadvertently influence the voluntariness of consent (Adhikari et al., 2018). Researchers should be cautious to ensure that building trust through social relationship does not endanger the voluntariness of participation (Geissler et al., 2008).

### Determining appropriate benefits; minimizing risk, burdens and exploitation

Community engagement can help to ensure that researchers provide appropriate benefits to the community – such as reimbursement and compensation for the time demands of study participation – and that participants are not unduly burdened (King et al., 2014). Ethics committees are tasked with reviewing applications to preclude research that is exploitative. However, their members may not be cognizant of local issues relevant to specific communities. Discussions with diverse stakeholders can be used to identify pertinent interests, including real and perceived risks and burdens that are often not apparent to outside observers. Based on the findings, engagement (and study activities) can be adapted to mitigate those risks (King et al., 2014).

Compensation and incentives must be tailored to the particular setting because of their potential to cause undue inducement (Emanuel, Currie, Herman, & Project, 2005; Gelinas et al., 2018). Community engagement, including discussions with community representatives and community members, can help researchers understand locally appropriate compensation and incentives (Adhikari et al., 2018; Participants in the Community Engagement and Consent Workshop, Kilifi, Kenya, March 2011, 2013). For instance, in a study of mass antimalarial drug administration (MDA) in Laos, community members advised the research team to provide non-monetary incentives, such as cooking utensils to participating households, rather than monetary incentives (Adhikari et al., 2017; Adhikari et al., 2017; Adhikari et al., 2018).

In remote communities, providing basic health care may be a prerequisite for commencing a study (Lavery, 2004; van Delden & van der Graaf, 2017) and this can have critical implications for researchers and the local population (Adhikari et al., 2016). For instance, researchers may be obliged to address common illnesses that communities prioritize and community members may not participate in a study if their needs are not met. Investing in basic healthcare infrastructure can have important budgetary and logistical implications and it is critical for funders to be aware that some studies in low- and middle-income countries need funding to be allocated to meet community health care needs.

Community engagement can also bring broader benefits, such as building health or research literacy, and improving basic infrastructure (van Delden & van der Graaf, 2017). For instance, in the MDA study in Laos, community volunteers were trained to provide information on study procedures, and broader education on malaria, preventive measures and general health and hygiene issues to their community (Adhikari et al., 2017). In addition, through active engagement, the study team became cognizant of poor potable water supply in the villages. Water pumps were therefore installed in each village to supply everyone with potable water (Adhikari et al., 2017).

### Supporting the consent process

Valid informed consent is a critical element of ethical health-related research. Valid consent entails (1) providing potential participants with adequate information about the proposed study; (2) potential participants understanding what is proposed and (3) participants being able to make a voluntary decision to participate (Nuffield Council on Bioethics, 2000). There are several ways in which community engagement can promote valid consent (Participants in the Community Engagement and Consent Workshop, Kilifi, Kenya, March 2011, 2013).

Community engagement can make consent more valid by enhancing the comprehensibility of the study concept and rationale (Bull & Lindegger, 2011; Participants in the Community Engagement and Consent Workshop, Kilifi, Kenya, March 2011, 2013). Insights gained from community engagement can help researchers decide what materials to use, how to provide information and how to tailor the information according to language, literacy and cultural protocols. For instance, the *Picture Talk Project* among aboriginal communities of the Fitzroy Valley in Australia revealed a preference for pictorial rather than written descriptions to explain the study rationale and objectives (Fitzpatrick et al., 2017). Effectively communicating information about research involves going beyond a literal translation of the consent form or information sheet and includes using locally appropriate analogies (Rubincam, Lacombe-Duncan, & Newman, 2016), pictures and demonstration material (Slack et al., 2016). This can cause conflict with the ethics committee and regulatory requirements, which may insist that formal documents are read to participants. Ethics committee members and regulatory authorities therefore must be engaged in dialogue to achieve an acceptable compromise.

Valid consent also requires voluntariness – intention and freedom from control from external factors (Bull & Lindegger, 2011). To maximize voluntariness, researchers should ensure that appropriate benefits are offered: too much and participants may be unduly influenced, too little and participants will be exploited (Bull & Lindegger, 2011). Community engagement can help researchers to identify social and economic factors that can affect voluntariness and uncover ways to mitigate them (Adhikari et al., 2017; Participants in the Community Engagement and Consent Workshop, Kilifi, Kenya, March 2011, 2013).

### Understanding vulnerabilities and researcher obligations

The vulnerability of potential research participants was traditionally addressed through labelling an entire class of individuals, such as pregnant women, children and ethnic minorities, as vulnerable and excluding them from study participation (ICH Harmonized Guideline: Integrated Addendum to ICH E6 (R1), 2016; Mechanic & Tanner, 2007; Luna, 2009). New ethics guidelines and many ethics scholars have challenged this approach: vulnerability is not inherent in an individual or groups but arise from social, economic and political conditions (CIOMS: International Ethical Guidelines for Health-Related Research Involving Humans, 2016; Luna, 2009) and the systematic exclusion from participation in research has resulted in a lack of evidence-based treatment for particular groups. Community engagement can help identify specific characteristics that may render individuals vulnerable, and this can aid in identifying special protections and researcher obligations needed for these individuals to be included in studies (Marsh, Kamuya, Rowa, Gikonyo, & Molyneux, 2008; Mechanic & Tanner, 2007; Mills et al., 2005). Engagement can also identify individuals’ capabilities, agency and resilience.

Although community engagement can help identify specific vulnerabilities, there can be inadvertent consequences of community engagement (Masquillier, Wouters, Mortelmans, & le Roux Booysen, 2015). For example, activities that single-out or offer benefits, which others perceive as excessive, to hard-to-reach groups can compound stigma related to particular diseases, such as HIV or tuberculosis (Nyblade, Singh, Ashburn, Brady, & Olenja, 2011). Time must therefore be invested to monitor the wider community response to engagement activities that target specific groups.

### Gaining permissions, approvals and building legitimacy

Community engagement can help to gain permissions, approvals and legitimacy for a planned study (King et al., 2014). Legitimacy is *formally* obtained through ethics committee approvals and permissions from local authorities or government in some instances. In some cases, even though formal approvals have been obtained, approvals and buy-in from influential stakeholders, such as politicians, and religious leaders who may not be directly involved in the study, are essential (Peto et al., 2018).

To gain permissions from national, regional and local authorities, researchers have to navigate the political system, hierarchical policy and the practice (Kaehler et al., 2019). Although securing permissions from the state are the foremost steps, initiating a study with approvals from the government/authorities alone can potentially have negative repercussions (Peto et al., 2018). For example, in politically divided communities in villages along the Thai-Myanmar border where anti-government movements are rooted in past conflicts and ongoing political opposition, gaining approvals from the government or one faction of the community hindered study recruitment (Kajeechiwa et al., 2017; König et al., 2018; Parker, Carrara, Pukrittayakamee, McGready, & Nosten, 2015). The perceived affiliation of the research team with a local political faction fuelled suspicion and opposition to the study. Cautious in-depth engagement with all stakeholders – including political and, religious leaders is needed, preferably during the research protocol development (Lavery, 2018).

Study protocols generally allow little flexibility in terms of procedures and timeline. Yet, with varied interests, participants or authority figures can make requests (for example, to improve local roads, change study procedure, such as ceasing blood draws) that are difficult to reconcile with the limits of the protocol (Lavery, 2018). Community engagement should ideally mean that proposed research is discussed as early as possible, and that what is at stake for different actors is addressed (Adhikari et al., 2017; King et al., 2014).

When seeking formal permissions, approvals and building legitimacy, focused and in-depth engagement is needed. One option is a deliberative approach that entails reaching a consensus while acknowledging differing views (King et al., 2014). Power differentials among stakeholders mean that building a consensus is often not a neutral process. Community engagement practitioners therefore require adequate preparation, experience and time to deal with this often challenging process. In our experience, study protocols do not always allocate sufficient funds or time for this. Moreover, full deliberation requires all community members to be able to contribute meaningfully to the discussions or decision-making process (Abelson et al., 2003). Although crucial to promote legitimacy, to ensure that all community members make meaningful contributions in deliberative approaches, those who have little experience of research or when they are unfamiliar with the study topic, they require specific support.

### Achieving recruitment and retention targets

Achieving recruitment and retention targets is crucial to maximize a study’s intended social value (Cheah & White, 2016). Failing to do so usually means that the study is underpowered or prematurely terminated. This is potentially a waste of resources and puts enrolled participants at risk for no good reason.

For example, large community-based studies, such as those of MDA require the participation of a large proportion of the community (Adhikari et al., 2016; Cheah & White, 2016; Habib et al., 2017; Newby et al., 2015; Pell et al., 2019; Williams et al., 2016). Achieving high population coverage is imperative in determining the effectiveness of MDA to interrupt local malaria transmission (Adhikari et al., 2016; Cheah & White, 2016; von Seidlein & Dondorp, 2015). In randomised controlled trials, achieving recruitment targets is important for the statistical power of these studies (Fletcher, Gheorghe, Moore, Wilson, & Damery, 2012). Although sometimes difficult to disentangle from the range of factors that influence recruitment and retention in a study, community engagement has been identified as improving recruitment and retention in several studies (Adhikari et al., 2016; Habib et al., 2017; Pell et al., 2019).

## Conclusion

Community engagement is increasingly promoted in global health research. Drawing on our experience of engaging communities in low- and middle-income settings – as programme-wide initiatives (König et al., 2018; Tangseefa et al., 2018), or alongside specific studies, such as MDA research (Adhikari et al., 2017; Adhikari et al., 2017; Adhikari et al., 2018; Kajeechiwa et al., 2017; Peto et al., 2018; Peto et al., 2018) and on others’ conceptual and empirical work (Dickert & Sugarman, 2005; Emanuel et al., 2004; Lavery, 2004; Lavery, 2018), this article describes how community engagement has the potential to complement and enhance established procedures – e.g. independent review and informed consent – to promote ethical good practice in research (Emanuel et al., 2004). Inevitably, this is unlikely to be an exhaustive description, particularly because community engagement experience and evaluation are often not shared outside particular research teams.

To achieve this potential, there are clear challenges to overcome and pitfalls to avoid. Commitment is needed by researchers and funders. Human resources and financial support are paramount to better embed community engagement in research practice. There is a danger of over-reliance on community engagement to ensure ethical good practice and assuming that it is a panacea for all research challenges. More empirical evidence is needed to demonstrate the contribution of community engagement to ethical good practice and to guide its planning, design and implementation (Lavery, 2018).

This article outlines seven overarching and interlinked areas in which community engagement can contribute to ethical health research: (1) respecting individuals, communities and stakeholders; (2) building trust and social relationships; (3) determining appropriate benefits; minimizing risks, burdens and exploitation; (4) supporting the consent process; (5) understanding vulnerabilities and researcher obligations; (6) gaining permissions, approvals and building legitimacy and (7) achieving recruitment and retention targets.
